# Dietary Intervention Impacts Immune Cell Functions and Dynamics by Inducing Metabolic Rewiring

**DOI:** 10.3389/fimmu.2020.623989

**Published:** 2021-02-04

**Authors:** Takuma Okawa, Motoyoshi Nagai, Koji Hase

**Affiliations:** ^1^ Division of Biochemistry, Faculty of Pharmacy and Graduate School of Pharmaceutical Science, Keio University, Tokyo, Japan; ^2^ Department of Gastroenterology, Research Center for Hepatitis and Immunology, Research Institute, National Center for Global Health and Medicine, Chiba, Japan; ^3^ International Research and Developmental Center for Mucosal Vaccines, The Institute of Medical Science, The University of Tokyo, Tokyo, Japan

**Keywords:** dietary intervention, calorie restriction, fasting, mTOR, AMPK, GCN2, metabolic rewiring

## Abstract

Accumulating evidence has shown that nutrient metabolism is closely associated with the differentiation and functions of various immune cells. Cellular metabolism, including aerobic glycolysis, fatty acid oxidation, and oxidative phosphorylation, plays a key role in germinal center (GC) reaction, B-cell trafficking, and T-cell-fate decision. Furthermore, a quiescent metabolic status consolidates T-cell-dependent immunological memory. Therefore, dietary interventions such as calorie restriction, time-restricted feeding, and fasting potentially manipulate immune cell functions. For instance, intermittent fasting prevents the development of experimental autoimmune encephalomyelitis. Meanwhile, the fasting response diminishes the lymphocyte pool in gut-associated lymphoid tissue to minimize energy expenditure, leading to the attenuation of Immunoglobulin A (IgA) response. The nutritional status also influences the dynamics of several immune cell subsets. Here, we describe the current understanding of the significance of immunometabolism in the differentiation and functionality of lymphocytes and macrophages. The underlying molecular mechanisms also are discussed. These experimental observations could offer new therapeutic strategies for immunological disorders like autoimmunity.

## Introduction

Abnormal nutritional conditions, such as malnutrition and diet-induced obesity, considerably affect the immunological status of the body. For instance, undernourished children are highly susceptible to infectious diseases and frequently show insufficient vaccine efficacy. In addition, multiple cohort studies have indicated that overnutrition and the prevalence of a westernized diet are associated with an increased incidence of inflammatory disorders, namely, metabolic syndrome, type 2 diabetes, allergy, and autoimmune disorders. Thus, diet and nutritional status significantly influence immune response. Furthermore, accumulating evidence has shown the importance of cellular metabolism in many aspects of immune cell biology. Inflammation-related M1 macrophages rely mainly on glycolysis, while immunosuppressive M2 macrophages utilize fatty acid oxidization (FAO) (1, 2). Naïve B and T lymphocytes are characterized by quiescent cellular metabolism, mainly depending on mitochondrial respiration (3, 4). Conversely, upon antigen recognition, naïve lymphocytes undergo metabolic rewiring to aerobic glycolysis to acquire effector functions. The amount of ATP production by glycolysis is much lower than that produced by mitochondrial respiration. Nevertheless, activated lymphocytes and macrophages exploit the prompt generation of ATP by glycolysis to fulfill the metabolic requirements for their proliferation and its provision of intermediate metabolites essential for the biosynthesis of nucleic acids, amino acids, and fatty acids.

Nutrient sensing is essential for the survival of cells and whole organisms, and thus the machinery of nutrient sensing and the downstream responses are highly preserved among eukaryotes (5). Cellular metabolic status is thus markedly affected by the extracellular milieu. For example, colonocytes at the luminal surface and upper crypts utilize short-chain fatty acids (e.g., butyrate), which is abundant in the colonic lumen, as a major energy source (6). In addition, white adipose tissue (WAT)-resident memory T cells and group 2 innate lymphoid cells (ILC2s) enhance FAO when activated (7, 8). Dietary intervention such as fasting prominently changes energy metabolism systemically, which in turn affects the immune cell biology, including cellular metabolism, cell dynamics, and survival. These findings raise the possibility that dietary intervention could offer novel approaches to ameliorate inflammatory disorders by modulating the immune response through metabolic rewiring. To date, a variety of dietary intervention protocols including calorie restriction, fasting, and nutrient supplementation have been proposed to regulate body weight, aging, the intestinal barrier, specific immune functions, and cognitive ability. In this review, we discuss the immunological significance of dietary intervention as well as the underlying mechanisms at both molecular and cellular levels. We also explore immunometabolism from a clinical perspective.

## The Impact of Dietary Intervention on Immune Responses

In the modern age, the food supply is generally stable thanks to agriculture and animal husbandry. Therefore, people residing in developed countries rarely face famine. Overnutrition has become a global health concern as a factor conferring a predisposition to metabolic syndrome, cancer, allergic diseases, and autoimmune disorders. Adjustment of dietary intake, frequency of meals, and dietary composition has emerged as a potential option to protect against the development of these diseases. However, recent studies have also demonstrated that certain intervention protocols occasionally lead to adverse events, such as the development of metabolic disorders (9, 10). Therefore, it is important to clarify the immunological consequences and underlying molecular mechanisms of various types of dietary intervention to maximize beneficial effects. In this section, we focus on four major types of dietary intervention in the context of immune–metabolic interaction.

### Calorie Restriction

**Figure 1:**
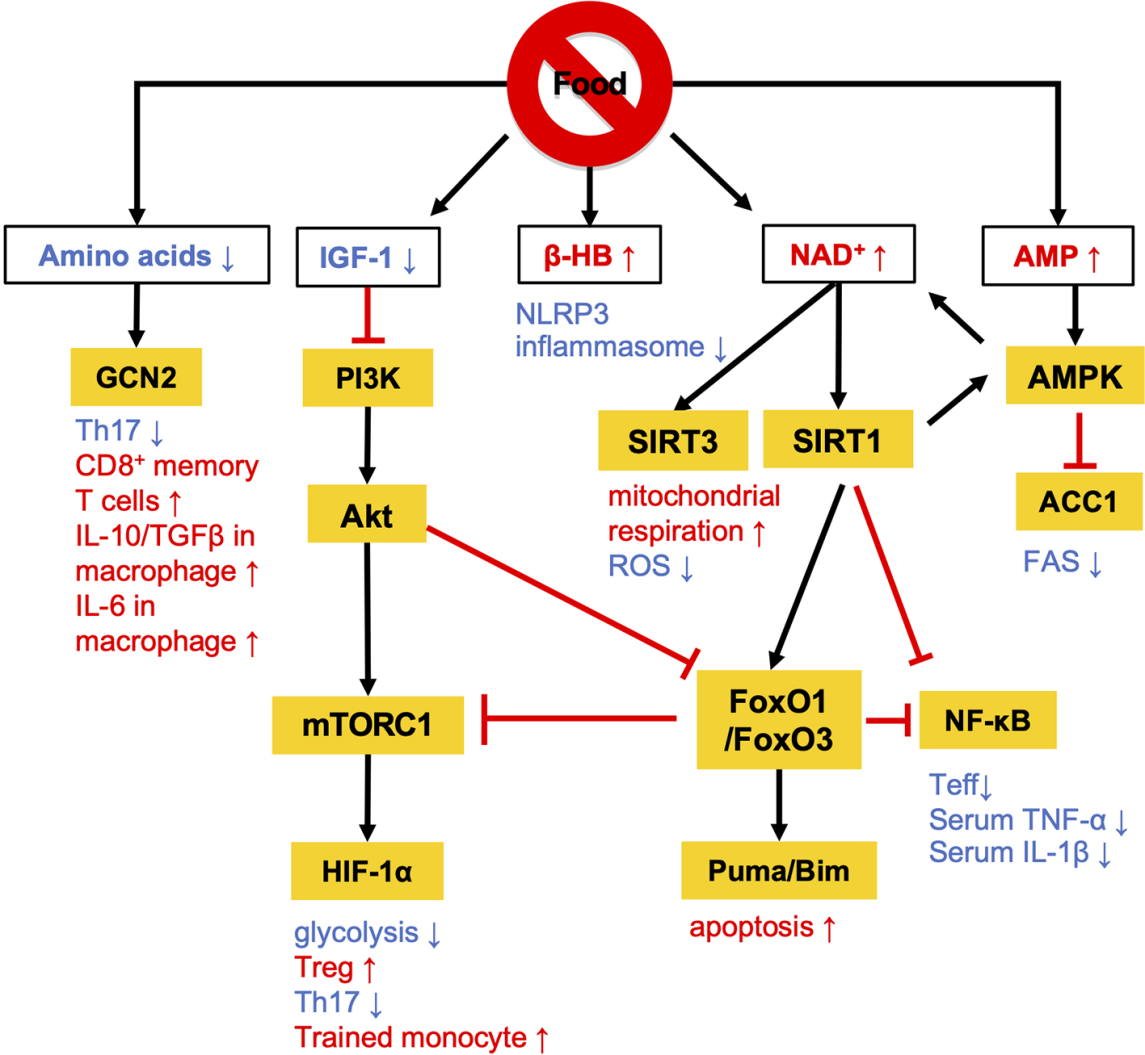
Overview of the nutritional signals regulating immune responses. Calorie restriction (CR) and fasting lowers plasma IGF-1 levels and downregulates PI3K/Akt/mTOR signaling pathways. At a low-energy status, two major energy sensors: adenosine monophosphate-activated protein kinase (AMPK) and sirtuin 1 (SIRT) family proteins, are activated by AMP and NAD^+^, respectively. GCN2 acts as a sensor of amino acid deficiency to regulate the differentiation and polarization of T cells and macrophages. β-HB also contributes to the anti-inflammatory effects by suppressing NLRP3 inflammasome activation. The white and orange boxes represent signal messengers and enzymes/transcription factors, respectively. The pathways depicted by black arrows and red bars represent the activation and inhibition by dietary restriction, respectively.

Calorie restriction (CR) without malnutrition involves a chronic reduction of energy intake by 15% to 40% compared with *ad libitum* conditions while maintaining an adequate intake of micronutrients such as vitamins and minerals. The link between CR and immunological functions was revealed nearly half a century ago (11). Since then, a number of studies have proven that CR suppresses the development of multiple diseases, such as cardiovascular disease, diabetes, cancer, and autoimmune disorders in human disease models (12–15). The activation of effector T (Teff) cells and M1 macrophages is highly dependent on the phosphatidylinositol-3 kinase (PI3K)-Akt-mechanistic target of rapamycin (mTOR) signaling, and persistent activation of this pathway by overnutrition drives M1-skewed inflammation (**Figure 1**). Conversely, the low-energy status conferred by CR suppresses the PI3K/Akt/mTOR axis with reciprocal activation of adenosine monophosphate-activated protein kinase (AMPK) and sirtuin family proteins (16–18) (**Figure 1**).

**Figure 2:**
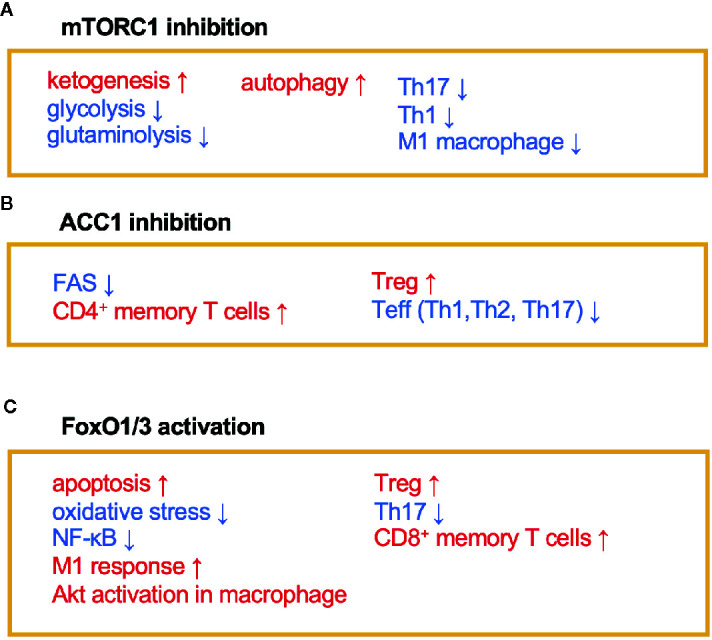
The immunomodulating effects of mTORC1, ACC1, and FoxO1/3. Fasting or calorie restriction (CR) suppresses mTORC1 and ACC1 activation and activates FoxO1/3 pathways. **(A)** mTORC1 inhibition enhances ketogenesis and reduces glycolysis and glutaminolysis. mTORC1 inhibition also induces autophagy in macrophage and suppresses Th1, Th17, and M1 macrophage differentiation. **(B)** ACC1 inhibition reduces FAS, which facilitates development of CD4^+^ memory T cells and Treg cells and conversely suppresses Teff (Th1, Th2, and Th17) responses. **(C)** Activation of FoxO1 and/or FoxO3 induces apoptosis and inactivates NF-κB. FoxO1 or FoxO3 also regulates phenotypes of macrophages, suppresses Th17 response, and induces development of CD8^+^ memory T cells and Treg cells.

AMPK inhibits the activity of acetyl-coenzyme A carboxylase 1 (ACC1), which leads to a reduction of fatty acid synthesis (FAS) (19) (**Figures 1** and **2**). The alteration of lipid metabolism is associated with the T-cell fate decision. For example, a 30% reduction of food intake for 4 weeks limited differentiation into Th17 cells and enhanced the development of regulatory T (Treg) cells by the inactivation of ACC1 in naïve T cells. Consequently, this treatment improved ischemic brain injury in a transient middle cerebral artery occlusion-induced ischemia model (20). Likewise, pharmacological inhibition of ACC1 by Soraphen A was reported to shape the Th17/Treg balance to improve the clinical score in an experimental autoimmune encephalomyelitis (EAE) model (21). The development of Th17 cells, but not Treg cells, requires ACC1-mediated *de novo* FAS. In addition, Th17 cells mainly utilize the glycolytic-lipogenic pathway to produce phospholipids for cellular membranes, whereas Treg cells actively take up exogenous fatty acids (21, 22). Furthermore, the other Teff cell subsets, such as Th1 and Th2 cells, rely on *de novo* FAS for their differentiation, and thus the inhibition of ACC1 can suppress their differentiation (21, 23). Conversely, ACC1 is dispensable for the activation of dendric cells and macrophages, even though *de novo* FAS is augmented upon mycobacterial infection (23). Therefore, ACC1 has emerged as a molecular target for drug development to regulate Teff cell-dependent inflammation.

A low-energy status during CR and fasting raises an intracellular level of nicotinamide adenine dinucleotide (NAD^+^) (24, 25). NAD^+^ is essential for glycolysis as well as oxidative phosphorylation (OXPHOS) as an electron transmitter. NAD^+^ also serves as a co-substrate for poly (ADP-ribose) polymerases (PARPs) and the sirtuin family (26–28). The intracellular NAD^+^ level is one of the critical determinants of differentiation and functions in macrophages. In human monocyte-derived macrophages, treatment with lipopolysaccharide (LPS) suppresses *de novo* NAD^+^ synthesis by inhibiting the kynurenine pathway and promotes the utilization of NAD^+^ by PARPs, lowering the intracellular level of NAD^+^ (29). Consequently, the LPS-induced decrease in NAD^+^ inactivates mitochondrial SIRT3, increases mitochondrial reactive oxygen species (ROS), suppresses mitochondrial respiration, and reciprocally activates glycolysis-dependent energy metabolism. Notably, such metabolic rewiring in response to the NAD^+^ levels substantially influences M1/M2 macrophage polarization. The augmentation of intracellular NAD^+^ by either overexpression of quinoline phosphoribosyltransferase (QPRT), a rate-limiting enzyme in the kynurenine pathway, or by supplementation of an NAD^+^ precursor rescues mitochondrial respiration in LPS-stimulated macrophages and thereby induces polarization to immunosuppressive M2 phenotype. Although the reduction of intercellular NAD^+^ pool facilitates differentiation into M1 phenotype, depletion of NAD^+^ leads to functional defect or reduces the viability of M1 macrophage due to the arrest of glycolysis (30). Indeed, NAD^+^ is integral to the simultaneous phosphorylation and oxidation of glyceraldehyde-3-phosphate to 1, 3-biphosphoglycerate, an essential step in glycolysis. Therefore, M1 macrophages utilize the NAD^+^ salvage pathway to maintain glycolysis and their functions, such as the production of pro-inflammatory cytokines (30). Inhibition of NAD^+^ salvage pathway by FK866 in LPS and/or IFN-γ-treated macrophages suppressed IL-1β and IL-6 production and decreased viability *in vitro*. Further, administration of FK866 ameliorated disease severity in an LPS-induced sepsis model.

Sirtuin 1 (SIRT1) is one of the NAD^+^-dependent deacetylases and serves as a significant regulator of metabolism and immune response. SIRT1 protein level increases in multiple cells and tissues in response to food deprivation and CR (31–33) (**Figure 1**). SIRT1 deacetylates nuclear factor kappa-light-chain enhancer of activated B cells (NF-κB), peroxisome proliferator-activated receptor γ (PPARγ), and hypoxia-induced factor 1α (HIF-1α) to regulate both innate and adaptive immune responses. In macrophages, deficiency in SIRT1 results in the hyperactivation of NF-κB, followed by the upregulation of pro-inflammatory cytokines such as TNF-α and IL-1β (34). In a syncytial virus infection model, the upregulation of SIRT1 in the lung was found to be essential to elicit respiratory immune responses and alleviate tissue damage (35). In this model, SIRT1 in dendritic cells promoted the production of Th1-inducing cytokines (e.g., IL-12 and TNF-α) and conversely suppressed the Th2 cytokines, leading to viral clearance and the resolution of inflammation. Furthermore, SIRT1 activation by NAD^+^ administration was found to ameliorate EAE symptoms by downregulating key transcription factors for Th1 (T-bet) and Th17 (RORγT and STAT3) (36). SIRT1 also inactivates HIF-1α through its deacetylating activity (37). Accumulating evidence has indicated the importance of HIF-1α for the Warburg-like metabolic rewiring toward aerobic glycolysis in immune cells. Therefore, SIRT1 may suppress the proinflammatory responses by inhibiting the HIF-1α-dependent metabolic rewiring (38). These findings raise the possibility that SIRT1 plays a role in the CR/fasting-dependent mitigation of inflammatory responses.

The immunoregulatory effect of CR may also be mediated by the Forkhead transcription factors O class 1 and 3 (FoxO1 and FoxO3) (**Figures 1** and **2**). Since CR alleviates PI3K/Akt signaling, which is a negative regulator of FoxO1 and FoxO3, CR eventually upregulates these transcription factors (39). In CD4^+^ T cells, FoxO1 and FoxO3 upregulate the expression of *Foxp3*, the master regulator of regulatory T (Treg) cells (40–42). Treg cells play a critical role in suppressing excessive immune responses by expressing immunosuppressive molecules (e.g., IL-10 and CTLA-4) and a high-affinity IL-2 receptor, CD25, which provokes IL-2 deprivation. T-cell-specific deletion of FoxO1 attenuates the TGF-β-induced differentiation of Treg cells (43). FoxO3 suppresses the proliferation and activation of Teff cells by inhibiting NF-κB (44), and also induces apoptosis by upregulating Puma and Bim (45). FoxO3-deficient mice showed spontaneous lymphoproliferation, associated with inflammation of the lung, kidney, and salivary gland (44). Such inflammation was found to correlate with the presence of hyperactivated Th1 and Th2 cells. In addition, mice carrying T cells deficient in FoxO1 and FoxO3 develop severe systemic autoimmune diseases mainly because of a defect in Treg cells and the activation of Teff cells (46). FoxO3 also suppresses the production of IL-6 from dendritic cells, which also contributes to the inhibition of Teff cells (47).

FoxO1 activation in macrophages exacerbates inflammatory responses. Exogenous expression of a constitutively active form of *Foxo1* in RAW264.7 cells potentiated LPS-induced TLR4 signaling pathway leading to phosphorylation of NF-κB, and vice versa, knockdown of *Foxo1* suppressed the TLR4 signaling pathway (48). Additionally, monocyte/macrophage-specific FoxO1-deficient mice (*LysM^cre/+^Foxo1^fl/fl^*) impaired TLR2-mediated response in liver-resident macrophages and failed to induce Th1 and Th17 response during *Staphylococcus aureus* infection (49). Interestingly, FoxO1-induced TLR2 and TLR4 signals induced Akt phosphorylation leading to FoxO1 inactivation. This negative feedback may constrain excessive inflammatory responses in macrophages and induce resolution of inflammation. Besides their immunomodulatory functions, FoxOs prevent tissue damage by suppressing oxidative stress and accelerating wound healing (50–53). This raises the possibility that CR-dependent activation of FoxOs may also promote wound healing, although further investigations are required to prove this.

In addition to anti-inflammatory effects, CR may consolidate immunological memory in response to vaccination. This possibility has been raised by findings from two recent independent studies: 1) 50% dietary restriction reinforced the functions of CD8^+^ memory T cells to protect against secondary bacterial infection and tumorigenesis (54) and 2) severe CR enhanced the proliferative response and cytokine production by T cells upon secondary infection with influenza (55). These effects by CR may be attributable to the inactivation of mTOR signaling that orchestrates glycolysis, glutaminolysis, and fatty acid biosynthesis (**Figures 1** and **2**). In support of this, low-dose treatment with an mTOR inhibitor, rapamycin, also facilitated the development and maintenance of memory T cells and conferred protection against viral infection (56). Similarly, *in vitro* culture of tumor-specific memory T cells under CR conditions enhanced anti-tumor functions, accompanied by the inactivation of mTOR signaling (57). Notably, ACC1 negatively regulates the transition of antigen-specific CD4^+^ T cells from effector to memory cell types (**Figure 2**). Therefore, genetic ablation or pharmacological inhibition of ACC1 enforces CD4^+^ memory T-cell formation in response to helminth infection (58). IL-7 is a cytokine essential for the survival of naïve and CD8^+^ memory T cells. Importantly, FoxO1 upregulates *Il7r* genes in these T-cell subsets to reinforce IL-7/IL-7R signaling, by interacting with other transcription factors: GABP and Gfi-1 (59, 60). Thus, CR is considered to strengthen the development and functionality of the memory T-cell subset by regulating multiple mechanisms. In addition to these molecular mechanisms in specific cell types, CR or fasting affects systemic metabolism, hormone release (e.g., insulin, glucagon, adipokines, and glucocorticoids), and nerve systems. Consequently, the pleiotropic effects of CR could be attributable to integration of the alterations of metabolic, endocrine, and nerve systems. Interestingly, the efficacy of vaccines is attenuated by both malnutrition and obesity (61–64), illustrating that an appropriate energy balance is a prerequisite to induce immune responses to vaccination fully.

### Fasting and Refeeding

Fasting refers to completely abstaining from food intake for certain periods ranging from several hours a day to a few weeks. There are many different fasting practices, including time-restricted feeding (TRF), intermittent fasting (IF), and periodic fasting (PF)/long-term fasting. Among them, TRF represents the daily restriction of food intake, usually for 12 to 20 h (65, 66). In IF, subjects or laboratory animals take little or no calories at least two days per week, but are allowed *ad libitum* feeding on the remaining days. IF protocols include alternative day fasting (ADF), in which 24-h fasting is repeated every other day (67, 68). Conversely, PF represents the intake of little or no calories for several days. PF has a more pronounced impact on metabolism and immune responses than CR or short-term fasting such as TRF and ADF (69).

Fasting has been performed as a religious practice. In Islamic tradition, abstinence from food and drink from dawn until sunset is encouraged during the month of Ramadan (70). Like CR, fasting also has a considerable impact on immune responses. IF during Ramadan results in significant decreases in circulating immune cells and pro-inflammatory cytokines (71, 72). Other studies have also demonstrated that Ramadan fasting induces the expression of antioxidant and anti-inflammatory genes in both nondiabetic obese patients and healthy subjects (73, 74). Fasting also reduces blood levels of glucose, insulin, insulin growth factor-1 (IGF-1), and amino acids, with the activation of AMPK and suppression of mTOR signaling. In response to these metabolic alterations, long-term hematopoietic stem cells undergo stress resistance, self-renewal, and regeneration (69). Fasting also induces whole-body FAO and ketogenesis in the liver to generate ketone bodies (i.e., acetone, acetoacetic acid, and β-hydroxybutyrate (β-HB)). In the kidney of aging-related chronic inflammation model, β-HB exerts an anti-inflammatory effect by activating FoxO1 through inhibition of Akt phosphorylation (75).

Furthermore, the refeeding phase in the fasting regimen may be necessary for cellular reprogramming and regenerative effects in various organs such as the liver, and gut (76). In an EAE model, IF was shown to ameliorate the disease symptoms by increasing the serum levels of adiponectin, corticosterone, and β-HB (77). Additionally, IF alters T-cell homeostasis in the gut with a decrease in Th17 cells and an increase in Treg cells. This effect is attributed to the alteration of gut microbiota by IF, which is characterized by an overrepresentation of Bacteroidaceae, Lactobacillaceae, and Prevotellaceae. The transplantation of fecal microbiota from IF mice was also shown to recapitulate EAE amelioration by IF (77).

Autophagy significantly contributes to somatic cell reprogramming and stem cell maintenance (78). The process of autophagy also serves as a protective factor against inflammation, infection, and neurodegenerative diseases (79–81). Autophagy was initially identified in *Saccharomyces* cultured under low-nutrient conditions (82). In the liver, farnesoid X receptor (FXR) and cAMP response element-binding protein (CREB), both of which are activated in response to nutrient signals, regulate the hepatic autophagy gene network (83). CREB was shown to promote the autophagic degradation of lipids under nutrient-deprived conditions, while FXR inhibited this response. Moreover, the CREB pathway enhanced alternatively activated M2 macrophage polarization in WAT (84). Furthermore, in the liver and muscle, refeeding after 24-h fasting suppressed autophagy by activating the mTOR complex 1 (mTORC1) pathway (85). Hence, fasting-induced autophagy might also lead to therapeutic effects.

TRF may also elicit its effect independent of the reduction of total calorie intake because TRF was shown to suppress weight gain and improve hyperinsulinemia, hepatic steatosis, and inflammation in mice fed a high-fat diet (HFD) (65). Notably, the total amounts of food intake were comparable between the TRF and *ad libitum*-fed groups. *Ad libitum* feeding with HFD disrupts the normal feeding cycle, with the mice eating the diet all day (86). Such feeding-cycle disruption is also prevalent in modern societies. This leads to the persistent activation of Akt/mTOR signaling as well as disturbance of the circadian oscillation of clock genes, both of which confer a predisposition to metabolic syndromes. TRF coordinates the balance of CREB, mTOR, and AMPK signaling and restores the circadian oscillations. The circadian oscillations are also observed in lymphocyte trafficking. At night, noradrenalin-dependent β2-adrenergic stimuli upregulate CCR7 and CXCR4 on B and T lymphocytes to suppress cell egress from the lymph nodes in mice (87). Additionally, the expression of sphingosine-1-phosphate receptor 1 (S1PR1), which also facilitates egress from the lymph nodes, by lymphocytes is also under the control of a circadian clock gene, Bmal1 (88). Expression of sphingosine-1-phosphate (S1P) is upregulated during the day. Consequently, CD4^+^ T cells accumulate in the lymph nodes at night and circulate during the day. In accordance with this observation, immunization with myelin-oligodendrocyte glycoprotein (MOG) at daytime augments the autoantigen-specific Th17 responses compared with nighttime immunization and exacerbates CNS inflammation in an EAE model (89). Taking these findings into account, the anti-inflammatory effect of TRF may partially be mediated by the normalization of the circadian oscillations.

Most previous studies have underscored the beneficial effects of calorie restriction and fasting on metabolic disorders and autoimmunity, where some reports showed adverse effects of such dietary interventions. The outcomes of calorie restriction and fasting are most likely dependent on feeding regimens, disease models, and the age of animals employed in each study. For example, a TRF in juvenile mice (4-8 weeks old) exacerbates metabolic disorders in adult age (12-week-old) (10). This study also manifested that TRF in juvenile mice affects sexual maturity as evidenced by retarded testicle development as well as high levels of serum GnRH, FSH, LH, and low levels of androgen and estrogen at 8-week-old. Such an observation is analogous to our findings that repeated fasting in juvenile mice (5-8 weeks old) attenuated the booster effect of oral immunization (90). Considering that GC reaction is essential for the induction of immunological memory, eliminating GC B cells from Peyer’s patches (PPs) by fasting may have resulted in this abnormality. This result is consistent with the observation of cohort studies that children with malnutrition fail to obtain vaccine efficacy (91–93). Thus, TRF and fasting in young adulthood may cause adverse effects in the metabolic, immunological, and enteroendocrine systems.

Recent clinical studies have shown the potential of a fasting-mimic diet (FMD), which is low in calories, sugars, and protein, but high in unsaturated fats, to achieve beneficial effects against aging, cancer, metabolic diseases, and cardiovascular diseases, in association with the reduction of body mass index, blood pressure and serum parameters (e.g., glucose, triglycerides, total and low-density lipoprotein cholesterol, IGF-1 and C-reactive protein) (94). Recent studies have also shown the effect of FMT on autoimmune diseases. Periodic 3-day FMD cycles ameliorated demyelination and symptoms in an EAE model (95). The 4-day cycles of FMD prevent the development of the dextran sodium sulfate (DSS)-induced colitis model by increasing the abundance of Lactobacillaceae and Bifidobacteriaceae with anti-inflammatory properties (96). Thus, FMD may be beneficial to improve both metabolic and inflammatory disorders.

### Specific Nutrient Restriction

Growing evidence has suggested that amino acid restriction (AAR) may be at least partly responsible for the immunomodulatory effect of dietary restriction. AAR inactivates mTOR signaling because certain amino acids, such as leucine, serve as activators of the mTOR/S6K1 pathway (97, 98). Leucine deprivation was found to improve insulin sensitivity of the whole-body and *in vitro-*cultured hepatocytes (99). In addition, a serine protein kinase, general control nonderepressible 2 (GCN2), functions as a sensor of amino acid deprivation. Leucine deprivation activates GCN2, which in turn inhibits mTOR signaling. Deficiency of GCN2 canceled the effect of leucine deprivation on insulin tolerance.

AAR also appears to regulate the differentiation and functions of immune cell subsets. For example, GCN2-dependent activation in response to AAR diminishes mouse and human Th17 differentiation (100). In an EAE model, GCN2-deficient mice showed severe disease symptoms even at the remission stage in association with increases in Th1 and Th17 response and a decrease in Treg cells (101). AAR also activates activating transcription factor 4 (ATF4) in CD4^+^ T cells. ATF4 transactivates a gene network that facilitates the amino acid intake and mTORC1 signaling (102). ATF4-dependent metabolic rewiring is required for the proliferation of CD4^+^ T cells and the development of Th1 cells.

Dietary tryptophan restriction (DTR) was found to impair the development of encephalitogenic Th17 cells to ameliorate EAE (103). Interestingly, GCN2 is dispensable for the effect of DTR, since GCN2-deficient mice fed a protein-free or tryptophan-free diet were reported to show resistance to EAE similar to that of WT mice. DTR suppresses gut inflammatory responses by shaping the gut microbial community. Furthermore, 40% dietary methionine restriction (DMR) was reported to reduce oxidative stress by suppressing the generation of mitochondrial ROS in rat heart (104). Oxidative stress is implicated in the development of chronic inflammatory disorders including inflammatory bowel disease (IBD). Therefore, DMR ameliorated the severity of DSS-induced colitis (105). DMR also delayed the senescence-associated secretory phenotype (SASP) in the kidney through hydrogen sulfide (H_2_S) generation and AMPK pathway activation (106).

GCN2 also contributes to the regulation of various macrophage functions. Among the major function of splenic macrophages is the clearance of apoptotic cells, leading to immune tolerance. Phagocytosis of apoptotic cells activated the indoleamine 2,3-dioxygenase 1 (IDO1)/GCN2 axis to upregulate IL-10 and TGF-β synthesis in splenic macrophages. Macrophages from monocyte/macrophage lineage-specific GCN2 knockout (*LysM ^cre/+^*/*Gcn2* ^fl/fl^) mice fails to acquire a tolerogenic phenotype (107). Backcross of *LysM ^cre/+^*/*Gcn2* ^fl/fl^ mice onto lupus-prone *FcgRIIB^-/-^* mice exacerbates systemic lupus erythematosus-like symptoms (107). Interestingly, tumor-associated macrophages from patients with melanoma also activate GCN2 and IL-10 production in the tumor microenvironment (108). Monocyte/macrophage-specific deletion of GCN2 drives the tumor-associated macrophages to induce antitumor responses. In contrast, GCN2 activation in RAW264.7 cells under tryptophan-free conditions results in the upregulation of inflammatory cytokines upon stimulation with LPS (109). The GCN2/eIF2/CHOP pathway mediates this response. The monocyte/macrophage-specific deletion of GCN2 improves the mortality after a lethal challenge with LPS by reducing the expression of IL-6 and IL-12 (109).

Carbohydrate restriction using a ketogenic diet (KD) also significantly regulates immune responses. KD is defined as a very low-carbohydrate, high-fat diet, which induces the generation of ketone bodies. A recent study revealed that KD alters gut microbiota. The KD-associated microbiota is characterized by an underrepresentation of *Bifidobacterium* spp. (110). β-HB produced by KD plays a central role in the decrease in *Bifidobacterium* spp., and this microbial environment prevents a pro-inflammatory Th17 response in the small intestine, but not the large intestine, of mice. However, it remains obscure how *Bifidobacterium* spp., which are usually located in the large intestine, induce the small intestinal Th17 cells. Notably, the reduction of bifidobacteria (e.g., *Bifidobacterium adolescentis* and *B. longum*) was also evident in humans fed KD for 4 weeks.

KD has been clinically used to cure intractable epilepsy, but the therapeutic mechanism behind this is largely unknown. Although the pathological mechanism has yet to be elucidated, an increased Th17/Treg cell ratio is considered to be a predisposing factor for intractable epilepsy (111). In young patients with intractable epilepsy, KD improved the imbalance of Th17/Treg cells in the blood through suppression of the mTOR/HIF-1 signaling pathway. Hence, KD may contribute to the treatment of intractable epilepsy by shaping the T-cell responses (111). Meanwhile, KD was found to induce the expansion of lung γδ T cells to strengthen epithelial barrier functions and antiviral resistance to influenza A virus (112). However, KD may exert distinct immunological effects depending on the duration of the treatment. Short-term (1-week-long) KD activates adipose-tissue-resident γδ T cells to support tissue repair, whereas long-term (4-month) KD suppresses this T-cell subset and exacerbates obesity in mice (113). Additionally, β-HB suppressed the NLRP3 inflammasome and attenuated the secretion of IL-1β and IL-18 in both mouse bone marrow-derived macrophages and human monocytes (114). The inhibition of the NLRP3 inflammasome is not dependent on AMPK, ROS, and autophagy, but is attributed to the prevention of K^+^ efflux and reduction of apoptosis-associated speck-like protein with a caspase recruitment domain (ASC) oligomerization.

Given that poor compliance and occasional adverse effects have limited the clinical application of CR and fasting, specific nutrient restrictions may be more feasible dietary interventions to achieve beneficial effects similar to those of CR and fasting.

### Nutrient Supplementation for the Regulation of Immune Response

Epidemiological evidence has suggested that malnutrition is a risk factor for infectious diseases and impairs vaccine efficacy (115–117). Vaccine efficacy depends on the formation of immunological memory. Vaccination evokes a GC reaction to generate plasma cells and memory B cells, which contribute to antibody production and long-lasting memory function, respectively. Anatomically, GCs are separated into the light zone (LZ) and the dark zone (DZ). GC B cells are highly mobile, circulating between the LZ and DZ (118). Upon antigen stimulation, naïve B cells migrate into the LZ, where the affinity-driven selection of GC B cells occurs through interaction with follicular helper T (Tfh) cells and follicular dendritic cells. The positively selected GC B cells activate mTORC1, which is required for migration into the DZ and vigorous proliferation (119). Several studies have demonstrated that the differentiation and the survival of Tfh cells and GC B cells highly depends on the mTOR signaling pathway (119, 120). We also confirmed that treatment with rapamycin greatly reduces the number of GC B cells in PPs (90). Deletion of Raptor or Rictor, which is the signature component of mTORC1 and mTORC2 respectively, in OX40^+^ cells (i.e. activated CD4^+^ T cells) impaired Tfh cell differentiation in PPs (120). In PPs, Tfh cells highly express glucose transporter Glut1 compared with other T-cell lineages to enhance glucose uptake. Glut1 expression in Tfh cells partly depends on mTORC1 signaling, since rapamycin treatment reduced Glut1 expression and glucose uptake. Nevertheless, GC B cells rely on FAO rather than glycolysis to fuel proliferation (121, 122); however, the mechanisms underlying how proliferating GC B cells actively oxidize fatty acids have remained unclear. Moreover, recent studies have indicated that several vitamins also participate in the regulation of B-cell homeostasis. Deficiency in vitamin B_1_ was found to decrease the number of naïve B cells in PPs. Vitamin B_1_-dependent maintenance of naïve B cells is required for the induction, but not effector, phase of the IgA response upon oral immunizaition (3).

Vitamin A (VA) deficiency is currently a global concern, especially in developing countries. Retinoic acid (RA), an active metabolite of VA, plays a central role in the lymphocyte trafficking to the gut, GC formation, and development of IgA^+^ plasma cells, leading to increased primary and secondary antibody responses (123, 124). Large peritoneal macrophages (LPMs), one of the mouse peritoneal macrophage subsets, supports IgA class-switching of peritoneal B-1 cells. RA-induced activation of GATA6 in LPM precursors causes their polarization and migration to the peritoneal cavity. In accordance with this, mice carrying monocyte/macrophage lineage-specific *Gata6* deletion (*LysM* ^Cre/+^/*Gata6* ^f/f^) or mice fed on VA-depleted diet (VAD) decreased the number of peritoneal macrophages, resulting in a decrease in B-1-derived IgA plasma cells in intestinal lamina propria. Thus, RA facilitates the differentiation of peritoneal B-1 cells into plasma B cells induced by LPMs (125). RA is also required for inflammatory resolution during helminth infection (126). *Schistosoma mansoni* infection transiently increases inflammatory F4/80^int^ CD206^+^ macrophages in the liver. This macrophage subset subsequently differentiates into F4/80^hi^ CD206^−^ macrophages to resolve acute inflammation. *S. mansoni*-infected mice fed on VAD were defective in the development of F4/80^hi^ CD206^−^ macrophages, leading to dysregulated inflammation and increased mortality.

Therefore, deficiency in RA attenuates the immune response and raises the risk of infectious diseases. To deal with VA deficiency, the World Health Organization has recommended high-dose VA supplementation in children 6–59 months of age in locations where VA deficiency is endemic. Oral supplementation of VA or RA potentiated vaccine efficacy by facilitating the trafficking of vaccine-antigen-specific T lymphocytes to the gastrointestinal mucosa in mice (127, 128). Moreover, the supplementation of VA or RA upregulated Stimulated by retinoic acid-6 (Stra6) in the spleen, enhancing anti-tetanus toxoid antibody production (129). Thus, VA supplementation has been considered a promising strategy to reinforce the antigen-specific immune response upon vaccination.

## The Effect of Dietary Intervention on Cell Dynamics

**Figure 3:**
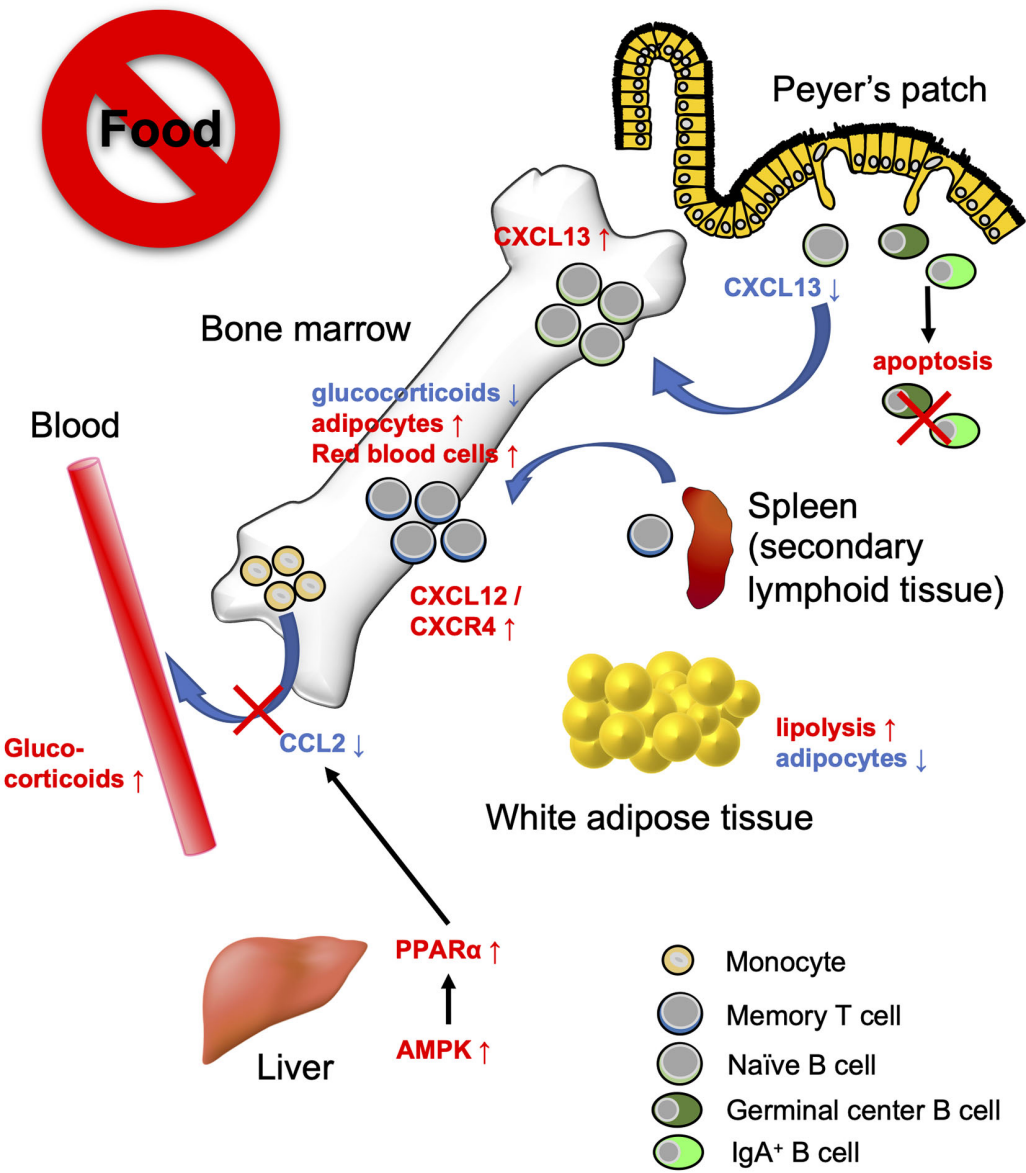
Bone marrow serves as a reservoir for several immune cell subsets in response to calorie restriction (CR) or fasting. Under low-energy conditions, naïve B cells, monocytes, and memory CD8^+^ T cells accumulate in the bone marrow. Fasting lowers CXCL13 levels in PPs and reciprocally increases the expression in the bone marrow. This leads to the migration of naïve B cells from PPs to the bone marrow. On the other hand, GC B cells and IgA^+^ B cells undergo apoptosis. Fasting diminishes circulating CCL2 levels through AMPK/PPARα signaling activation. Consequently, the egress of monocytes from the bone marrow is suppressed. Dietary restriction drives CD8^+^ memory T cells to traffic toward the bone marrow in the S1P/S1PR1- and CXCL12/CXCR4-dependent manner. The bone marrow microenvironment provides a tissue-specific niche for the maintenance of memory CD8^+^ T cells with low glucocorticoid concentration and abundant adipocytes.

Recent studies have revealed that the bone marrow serves as a reservoir for naïve B cells, monocytes, and memory CD8^+^ T cells in response to a low-energy status like that found in CR and fasting (54, 90, 130) (**Figure 3**). These findings indicate that nutritional status influences the cell dynamics of several immune cell subsets. We observed that naïve B cells migrated from PPs to the bone marrow in a CXCL13-dependent manner during 36 h of fasting in mice (90). CXCL13 expression by stromal cells is essential for the formation and maintenance of lymphoid follicles in the lymphoid organs including PPs (131, 132). The *Cxcl13* mRNA expression was significantly downregulated in PPs during the fasting period. *In vitro* study using a lymph node-derived stromal cell line suggested that cytokine stimulus (i.e., TNF-α and LTβR agonists)-dependent upregulation of *Cxcl13* is accompanied by an increase in glycolysis. Because treatment with a glycolysis inhibitor, 2-deoxyglucose, mitigated *Cxcl13* expression by activated BLS12 cells in a dose-dependent manner, the metabolic rewiring to glycolysis plays a pivotal role in *Cxcl13* expression. The 36 h of fasting markedly lowered blood glucose levels and attenuated glycolysis, resulting in the downregulation of *Cxcl13* expression. In sharp contrast, in the bone marrow, *Cxcl13* expression significantly increased during fasting, which allowed naïve B cells to accumulate mainly in the vicinity of blood vessels of the bone marrow cavity. Such a perivascular region is also known as a site for hematopoietic stem cell (HSC) differentiation and proliferation due to the accumulation of survival factors such as B-cell activating factor (BAFF), colony-stimulating factors (CSF), and stem cell factors (SCF), which are most likely fundamental for the survival of B cells (133–135). Therefore, the perivascular region could serve as a transient niche for naïve B cells during fasting. In response to refeeding, *Cxcl13* expression in the bone marrow was downregulated and naïve B cells migrated back to PPs. Thus, the number of naïve B cells was gradually restored until 48 h after refeeding. In contrast, the recovery of GC B cells and IgA^+^ B cells was much slower than that of naïve B cells because these cells readily underwent apoptosis in response to fasting. As a consequence, GC B cells were eliminated from lymphoid follicles of PPs, whereas naïve B cells were restored after fasting-refeeding. Give that a subset of GC B cells differentiates into memory B cells, the elimination of GC B cells from PPs in fasted mice may lead to the loss of immune memory for oral antigens. Indeed, fasted mice failed to generate antigen-specific IgA, IgM, and IgG upon repeated oral immunization with ovalbumin.

Jordan *et al.* found that short-term (4–20 h) fasting suppressed the CCL2/CCR2 axis, which is essential for the egress of inflammatory monocytes from the bone marrow into the bloodstream (130). This short-term fasting activated AMPK/PPARα signaling in hepatocytes to lower the level of circulating CCL2. Although monocyte dynamics are also regulated by the CXCL12/CXCR4 axis in the bone marrow, the CXCL12 expression level was not affected by fasting (136). Fasting-induced accumulation of monocytes in the bone marrow was independent of fibroblast growth factor 21 (FGF21) and ketone body production. Transcriptome analysis demonstrated that monocytes were in a quiescent state during fasting. Notably, upon infection with *Listeria monocytogenes*, monocytes normally migrated from the bone marrow into the bloodstream and eliminated the pathogen without causing inflammation.

Collins *et al*. showed that 50% dietary restriction promoted the accumulation of circulating CD8^+^ memory T cells in the bone marrow in an S1P/S1P receptor 1- and CXCL12/CXCR4-dependent manner (54). CD8^+^ memory T cells resided in WAT under physiological conditions; however, they preferentially migrate to the bone marrow at low-energy status. Under CR conditions, the concentration of glucocorticoid is increased in the blood but decreased in the bone marrow. Because a high concentration of glucocorticoid induces apoptosis in memory CD8^+^ T cells, these cells can survive in the bone marrow under CR conditions. Furthermore, dietary restriction led to the differentiation of adipocytes that generate fatty acids. Memory CD8^+^ T cells actively utilize fatty acids for mitochondrial FAO (137–139). Thus, the bone marrow microenvironment provides a niche for memory CD8^+^ T cells under the low-energy conditions (54). It is worth noting that dietary restriction was also found to enhance the protective function of memory T CD8^+^ cells; dietary-restriction promoted clearance of influenza and *Yersinia pseudotuberculosis* in mice.

These recent studies revealed the function of the bone marrow as a shelter for several immune cell subsets during metabolic adversity. Such a multiorgan-trafficking of immune cells may occur even under normal nutritional conditions. In support of this notion, we observed that naive B cells show circadian oscillation between the bone marrow and PPs. The number of naive B cells in the bone marrow increased during the daytime when mice usually take little food, whereas the number decreased during nighttime in response to food intake. Furthermore, FoxO1, which is upregulated during CR, regulates naïve T cell migration to the secondary lymphoid tissues by increasing the expression of CCR7 and L-selectin. We are only beginning to learn about the multiorgan-trafficking of immune cells as a fasting response. Further investigation will clarify the underlying mechanisms in this emerging field.

## A Clinical Perspective on Immunometabolism

In addition to dietary interventions, drugs targeting key nutrient signaling pathways (e.g., AMPK and mTOR) have attracted substantial attention in efforts to improve metabolic and inflammatory disorders. Rapamycin is best characterized as an inhibitor of mTORC1, and its chronic administration also inhibits mTORC2 in some tissues. Rapamycin has been clinically used as an immunosuppressant to prevent post-transplantation rejection. Animal studies have also corroborated that rapamycin-dependent mTOR inhibition is effective for various diseases, including ischemic stroke, rheumatoid arthritis, and EAE (140–143) (**Table 1**). The anti-inflammatory effect of rapamycin treatment is associated with the attenuation of Th17-cell differentiation as well as the promotion of Treg-cell development (142, 143). Moreover, the coadministration of rapamycin with FMS-like tyrosine kinase 3 (FLT-3) ligand was shown to facilitate the plasmacytoid dendritic cell-dependent induction of Treg cells, thereby enhancing immune tolerance (144). Rapamycin was also reported to alleviate the development of a murine atherosclerosis model. During the initial phase of atherosclerosis, peritoneal macrophages transform into foam cells that elicit plaque formation inside the arterial vessels (145). Rapamycin was found to induce autophagy in macrophages to prevent foam cell development (146). However, rapamycin and everolimus, a derivative of rapamycin, possess only a narrow therapeutic index because of their pleiotropic effects, which potentially cause adverse effects.

**Table 1:** Drugs targeting the metabolic pathways of immune cells.

Drugs	Conventional application	Targeting metabolism	Immunological change	Tissue	New applications
Rapamycin (sirolimus) Everolimus	post-transplantation rejection suppression	Inhibition of mTORC1 (mTORC2)	autophagy in macrophages↑ Th1/Th17 ↓ Treg ↑ memory CD8^+^ T cells ↑	Brain arterial-vessels	rheumatoid arthritis EAE/MS ischemic stroke atherosclerosis viral infection
Metformin	type 2 diabetes	Inhibition of mitochondrial complex I Activation of AMPK Inhibition of mTOR	Inhibition of NLRP 3- inflammasome Th1/Th17 ↓ Treg/IL-10 ↑ IL-1β↓	brain lung liver intestine skin	IBD MS sepsis-induced acute lung injury psoriasis
Dimethyl fumarate	Psoriasis	Inhibition of GAPDH	Activation of macrophages↓ Th1/Th17 ↓ Treg ↑	spleen intestine brain	EAE
Mevalonate	a metabolite of cholesterol synthesis	Activation of mTOR-HIF-1 pathway	trained monocyte ↑	(blood)	innate immune memory ↑ cancer
Soraphen A	Fungicide	Inhibition of ACC1	Inhibition of FAS Th17 ↓ Treg ↑	brain	ischemic stroke
Halofuginone	the plant alkaloid Dichroa-febrifuga	Inhibition of prolyl-tRNA synthetase Activation of GCN2	Th17 ↓ GC formation ↑ memory CD8^+^ T cells ↑	intestine brain	colitis MS vaccine efficacy↑

Metformin, the first-line drug for type 2 diabetes, has the potential to regulate immune responses through inducing metabolic rewiring in immune cells such as T cells (**Table 1**). It inhibits mitochondrial complex I and restrains hepatic gluconeogenesis, with an increase in glucose utilization in peripheral tissues (147). Such inhibition of mitochondrial complex I was found to enhance hepatic AMPK, which in turn established a CR/fasting-like metabolic status (148). A low concentration (0.5 mM) of metformin inhibited hepatic mTORC1 through the AMPK-related pathway in mice (149). Similar to dietary interventions, metformin and a complex I inhibitor, rotenone, were reported to suppress mitochondrial ROS generation and NLRP3-dependent inflammasome formation in animal and human studies (150–152). Accordingly, metformin treatment decreased IL-1β production while increasing IL-10 in response to LPS (153). Metformin treatment also suppressed the development of Th17 cells by inhibiting mTOR and STAT3 signaling, whereas it induced Treg cells by enhancing AMPK signaling (154). These studies demonstrate that metformin has a therapeutic effect on inflammation-related diseases, including IBD, EAE, rheumatoid arthritis, and psoriasis (154–158). These observations also raise the possibility that a combination of dietary intervention and metformin may induce synergy on the anti-inflammatory responses. In a preliminary clinical trial, a combination of a dietary restriction with metformin on obese people enforced protective effects on insulin resistance (159). Interestingly, a low concentration (10 µM) of metformin stimulated exhausted tumor-infiltrating CD8^+^ T cells to upregulate IL-2, TNF-α, and IFN-γ and potentiated anti-tumor activity (160). Metformin also diminished tumor-resident Treg cells that counteract anti-tumor immunity (161). Although Treg metabolism largely depends on OXPHOS through FAO, metformin-treated Treg cells undergo glycolysis. These facts indicate that low-dose metformin augments anti-tumor immunity by inducing metabolic rewiring.

Halofuginone (HF), a derivative of the plant alkaloid *Dichroa febrifuga* is a pharmacological mimic of AAR. HF competitively inhibited prolyl-tRNA synthetase, enhancing the intracellular pool of uncharged tRNA that phosphorylates and activates GCN2 (162). HF treatment selectively constrained Th17 differentiation, but not Th1, and protected the development of EAE (100). The administration of HF also inhibited IL-1β production by LPS-stimulated macrophages and ameliorated the severity of DSS-induced colitis (163). Additionally, HF promoted GC formation and memory B cell formation in the draining lymph nodes in mice received vaccination (164). HF treatment also increased antigen-specific effector CD4^+^ and CD8^+^ T cells upon the secondary stimulation. Based on these observations, the pharmacological mimic of AAR responses may be a promising strategy to dampen inflammatory response and consolidate immunological memory for vaccine antigens (**Table 1**).

Other drugs targeting the metabolic system have also become key options for immune modulation. Dimethyl fumarate (DMF), a derivative of the Krebs cycle intermediate fumarate (165). DMF inactivated a glycolytic enzyme, glyceraldehyde 3-phosphate dehydrogenase (GAPDH) and inhibited aerobic glycolysis in activated, but not resting, macrophages (**Table 1**). The inhibition of glycolysis by DMF limited differentiation and functions of CD4^+^ T cells cultured under Th1- and Th17-polarized conditions.

Innate immune cells also develop long-term memory upon stimulation with bacterial products like β-glucan. This phenomenon is termed as trained immunity (166). A metabolic shift from OXPHOS to aerobic glycolysis, namely, Warburg-like effect, is vital for β-glucan-induced trained immunity. Such a metabolic alteration is caused by the activation of mTOR-HIF-1 pathway (167). The induction of trained immunity is also dependent on mevalonate, a metabolite of cholesterol synthesis. Mevalonate not only active the mTOR-HIF-1 pathway but also cause epigenetic alternations characterized by enrichment of H3K4me3 on the promoter region of *IL6* and *TNFA* gene loci (168). Inhibition of mevalonate synthesis by fluvastatin canceled the increment of cytokine production as well as epigenetic alternations induced by either β-glucan or oxidized low-density lipoprotein in monocytes. Thus, cholesterol biosynthetic pathways are considered as a drug target to interrupt innate immune memory (**Table 1**).

## Conclusion

Dietary interventions have profound effects on immune responses through metabolic rewiring. Fasting has been shown to enhance immune memory and suppress inflammation. TRF was shown to recover the appropriate circadian rhythm, improving metabolic disorders and optimizing immune responses. Moreover, accumulating studies have demonstrated the molecular mechanisms underlying these findings. Thus, drugs targeting the metabolic system may become a critical option for immune modulation.

Meanwhile, dietary interventions of different types, durations, and timings can have opposite effects on health and disease. The benefits of dietary interventions can vary from patient to patient when we apply such interventions in a clinical setting. Experimental protocols have been performed to treat various immune-related disorders with dietary interventions. Looking ahead, we need to develop evidence-based, optimized protocols to avoid adverse effects. New therapeutic approaches against type 2 diabetes and cardiovascular disease are currently being discussed and applied (169–171). It is reasonable to use the same approach to treat inflammatory diseases and enhance vaccine efficacy.

In addition to local responses, an integrated immunometabolic response is required for host survival in a state of energy deficit. Recent studies have revealed the function of the bone marrow as a shelter for immune cells during dietary deficiency. It is clear that metabolic rewiring impacts immune cell dynamics, inducing inter-organ fasting responses in the body. A deeper understanding of this field could lead to the identification of novel drug targets.

## Author Contributions

TO and MN wrote the manuscript. TO prepared the figures. KH critically revised the manuscript and obtained funding. All authors contributed to the article and approved the submitted version.

## Funding

This study was supported by grants from the Japan Society for the Promotion of Science (20H00509 to KH) and AMED-Crest (20gm1010004h0105 and 20gm1310009h0001 and to KH).

## Conflict of Interest

The authors declare that the research was conducted in the absence of any commercial or financial relationships that could be construed as a potential conflict of interest.
